# The Effect of Virtual Endoscopy Simulator Training on Novices: A Systematic Review

**DOI:** 10.1371/journal.pone.0089224

**Published:** 2014-02-21

**Authors:** Weiguang Qiao, Yang Bai, Ruxi Lv, Wendi Zhang, Yuqing Chen, Shan Lei, Fachao Zhi

**Affiliations:** 1 Guangdong Provincial Key Laboratory of Gastroenterology, Department of Gastroenterology, Nanfang Hospital, Southern Medical University, Guangzhou City, Guangdong Province, China; 2 School of Traditional Chinese Medicine, Southern Medical University, Research Institute of Traditional Chinese Medicine, Guangdong Medical College, Zhanjiang City, Guangdong Province, China; University Hospital Llandough, United Kingdom

## Abstract

**Background:**

Advances in virtual endoscopy simulators have paralleled an interest in medical simulation for gastrointestinal endoscopy training.

**Objective:**

The primary objective was to determine whether the virtual endoscopy simulator training could improve the performance of novices.

**Design:**

A systematic review.

**Setting:**

Randomized controlled trials (RCTs) that compared virtual endoscopy simulator training with bedside teaching or any other intervention for novices were collected.

**Patients:**

Novice endoscopists.

**Interventions:**

The PRISMA statement was followed during the course of the research. The Cochrane Central Register of Controlled Trials, MEDLINE, EMBASE, and ScienceDirect were searched (up to July 2013). Data extraction and assessment were independently performed.

**Main outcome measurements:**

Independent procedure completion, total procedure time and required assistance.

**Results:**

Fifteen studies (n = 354) were eligible for inclusion: 9 studies designed for colonoscopy training, 6 for gastroscopy training. For gastroscopy training, procedure completed independently was reported in 87.7% of participants in simulator training group compared to 70.0% of participants in control group (1 study; 22 participants; RR 1.25; 95% CI 1.13–1.39; *P*<0.0001). For colonoscopy training, procedure completed independently was reported in 89.3% of participants in simulator training group compared to 88.9% of participants in control group (7 study; 163 participants; RR 1.10; 95% CI 0.88–1.37; *P = *0.41; *I* ^2^ = 85%).

**Limitations:**

The included studies are quite in-homogeneous with respect to training schedule and procedure.

**Conclusions:**

Virtual endoscopy simulator training might be effective for gastroscopy, but so far no data is available to support this for colonoscopy.

## Introduction

Gastrointestinal endoscopy, especially for gastroscopy and colonoscopy, is the basic skills required for gastroenterology fellows. While skills acquisition mainly depends on experience and practice, the traditional endoscopy training has largely involved observing and then performing the procedure on patients under the supervision of an expert endoscopist. There is an increasing concern that traditional endoscopy training methods are suboptimal for patient care. Novices are significantly more likely to encounter complications [1 2], patient discomfort [3] and long procedure time [4] than experienced endoscopists for diagnostic endoscopic procedure, let along therapeutic endoscopic procedures.

Recently, virtual endoscopy simulator training has been described as a potential substitute for conventional training [5]–[8]. Computer-based endoscopic simulators offers several potential advantages over traditional bedside teaching: (1) interactive video technology uses endoscopic images stored on a disk and could display them in real time in response to user’s endoscopic movements [9]; (2) computer graphics simulation uses computerized images displayed in response to the endoscopy being performed [10]; (3) no procedure-related errors to patients [11]. This systematic review was to determine whether the endoscopic skills of novices are improved after virtual endoscopy simulator training.

The purpose of this systematic review was to compare virtual simulator training with conventional teaching of gastrointestinal endoscopy. The hypothesis was that the virtual reality simulator, by virtue of the many potential advantages noted above, would be equal to or better than conventional teaching of gastrointestinal endoscopy to internal medicine and general surgical novices in both efficacy and safety.

## Methods

The protocol for this systematic review was registered on PROSPERO (CRD42013005695) and is available in full on the NIHR (National Institute for Health Research) website (http://www.crd.york.ac.uk/prospero/display_record.asp?ID=CRD42013005695 ) [12].

### Search Strategy

The PRISMA (Preferred Reporting Items for Systematic Reviews and Meta-Analyses) statement was followed during the course of this research. Bibliographical searches were performed up to July 2013 in the Cochrane Central Register of Controlled Trials, MEDLINE (1991 to July 2013), EMBASE (1991 to July 2013), and Scopus (ScienceDirect 1991 to July 2013) databases.

Search terms included:

- Endoscopy

EndoscopyFlexible endoscopyFiber endoscopyColonoscopyGastroscopyGastroenterological endoscopyGastrointestinal endoscopyUpper gastrointestinal endoscopyLower gastrointestinal endoscopyThese terms were combined through using the Boolean operator “OR”

- Simulator training

Simulator trainingSimulation trainingVirtual reality trainingVirtual reality curriculumVirtual reality simulationVirtual reality simulatorComputer based simulatorComputer based virtual reality simulatorsAccu Touch Endoscopy SimulatorGI mentor IIErlangen Active Simulator for Interventional Endoscopy (EASIE)Observational Learning

These terms were combined through using the Boolean operator “OR”. The results of searches a and b were combined through using the Boolean operator “AND”. Manual searches were conducted for eligible studies and abstracts from references of published articles. An expanded search was performed on Google Scholar, and authors were contacted for additional information when necessary.

### Definition

Virtual endoscopy simulator training was defined as that the novices received structure training schedule based on the virtual endoscopy simulator. No simulator training group, known as the control group, was defined as that the novices received bedside training or any other training rather than simulator training. Bedside teaching, also known as traditional training or conventional teaching, was based on hands-on procedures. Trainees passively observe examinations carried out by an expert and then begin to do examinations by themselves with the assistance of an expert. The other training methods mainly contain theoretical training and videos watching. Main outcome measurements were independent procedure completion, total procedure time and required assistance. All these outcomes were measured after training.

### Study Selection

To be eligible for inclusion in this systematic review, studies were required to meet the following criteria: (1) Randomized controlled trials (RCTs); (2) comparison between virtual endoscopy simulator training and bedside teaching any other intervention for novices were collected; (3) information on independent procedure completion, total procedure time or required assistance were provided; and (4) being published. When the same author reported results from the same patient population, the most recent or the most complete publication was included. The exclusion criteria were as follows: (1) study population or trial size was not clear; (2) non-RCT, qualitative study or study without extractable data; (3) qualitative study or study without extractable data; (4) published as a case report, editorial, commentary, review, or abstract only. There were no language or location restrictions. Study citations and abstracts were collected, and full papers were retrieved to screen for potentially relevant papers. The above procedures of literature search and article selection were independently performed by 2 authors (Weiguang Qiao and Ruxi Lv). All disagreements were resolved by consensus.

### Data Extraction

Two investigators (Wendi Zhang and Yuqing Chen) independently extracted the available data (independent procedure completion, total procedure time and required assistance), with discrepancies resolved by consensus. The following data were also collected for each study: first author of study, year(s) conducted/published, country and geographical region, number of study centers, simulator system, endoscopy type, baseline demographics about participants, treatment and control regimens.

### Risk of Bias Assessment

Two investigators (Weiguang Qiao and Ruxi Lv) independently evaluated the quality of the included studies by using the Application of Cochrane Collaboration’s tool for assessing risk of bias. Each study was assessed for random sequence generation, allocation concealment, blinding of participants and personnel, blinding of outcome assessment, incomplete outcome data, selective reporting, and other potential sources of bias. Each factor will be rated as “low risk” of bias (e.g., random sequence generation was computer generated), “high risk” of bias (e.g., participants and personnel were not blinded) or “unclear risk” of bias (e.g., methods used for allocation concealment were not described in the manuscript). Disagreements will be resolved by consensus.

### Statistical Analysis

The Cochrane Collaboration review manager RevMan (Version 5.1) was used for data analysis. Two investigators (Weiguang Qiao and Shan Lei) were involved in the statistical analysis. For binary outcomes, risk ratio (RR) and 95% confidence intervals (95% CI) were calculated based on a fixed-effect model or a random-effect model. For continuous outcomes, the standardized mean difference (SMD) and 95% CI were calculated based on fixed-effect model. The statistical significance of heterogeneity among studies was assessed by inspection of graphical presentations and by calculating the *chi* square test for heterogeneity (a *P-*value of 0.10 was regarded as statistically significant). To quantify the effects of heterogeneity, the *I* ^2^ statistic was used. Heterogeneity was considered significant if the *P*-values were 0.1 or less and *I* ^2^ was more than 50%. The fixed effect model was employed in pooling data where there was no evidence of heterogeneity and where there was evidence of heterogeneity, the random effects model was used instead when there was unexplained heterogeneity. A funnel plot was not conducted to investigate publication bias as there were too few studies included in each comparison to produce a meaningful analysis. Subgroup analysis was also carried out because the gastroscopy and colonoscopy were quite different.

## Results

### Characteristics of Individual Studies

**Figure 1:**
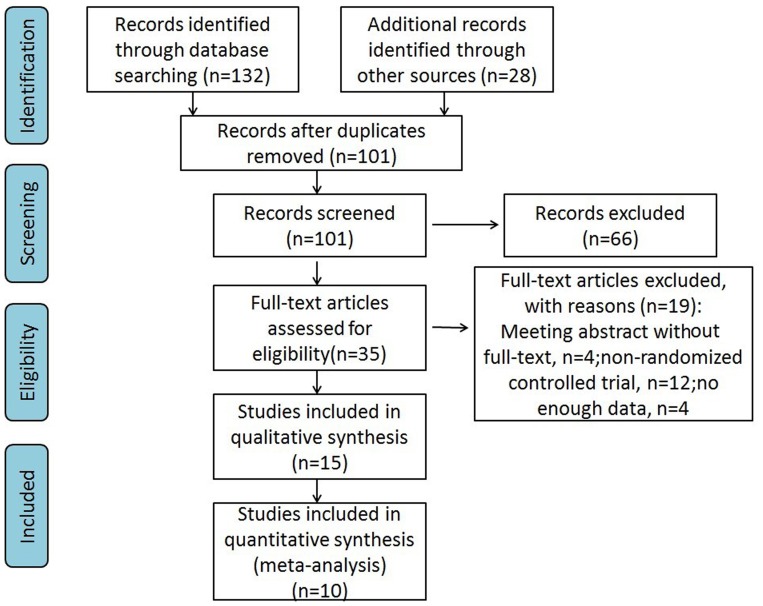
Flow diagram of study selection for the systematic review.

A total of 101 citations were identified after searching. Only 15 RCTs met the inclusion criteria listed in Table S1 [13]–[27]. A total of 19 articles were excluded (meeting abstract without full-text, n = 4; non-randomized controlled trial, n = 12; no enough data, n = 4). Most of the trials included in the analysis were conducted in USA (8 in USA, 4 in Germany, 2 in Italy, 2 in Austria, 1 each in Sweden, UK, Denmark, France, Canada and Netherlands). A total of 354 participants were included. Three studies (Cohen 2006, Haycock 2010, Maiss 2007) had a sample size larger than 30. Nine studies included were designed for colonoscopy training, while 6 for gastroscopy training. The simulator systems used include: the Accu-Touch colonoscopy simulator (Ahlberg 2005, Kruglikova 2010, Park 2007, Sedlack 2004), the GI Mentor virtual endoscopy simulator (Cohen 2006, Di Giulio 2004, Ende 2012, Ferlitsch 2002, Ferlitsch 2010), the Erlangen Active Simulator for Interventional Endoscopy (EASIE) (Ende 2012, Hochberger 2005, Maiss 2007, Maiss 2006), the simulated ScopeGuide 3-D imager view (Haycock 2010) and virtual reality sigmoidoscopy simulator (Gerson 2003, Tuggy 1998). The trial selection process is presented in Figure 1.

### Risk of Bias in Included Studies

**Figure 2:**
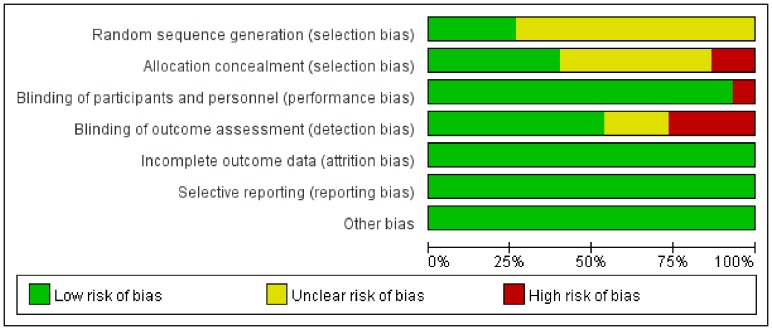
Risk of bias assessment.

**Figure 3:**
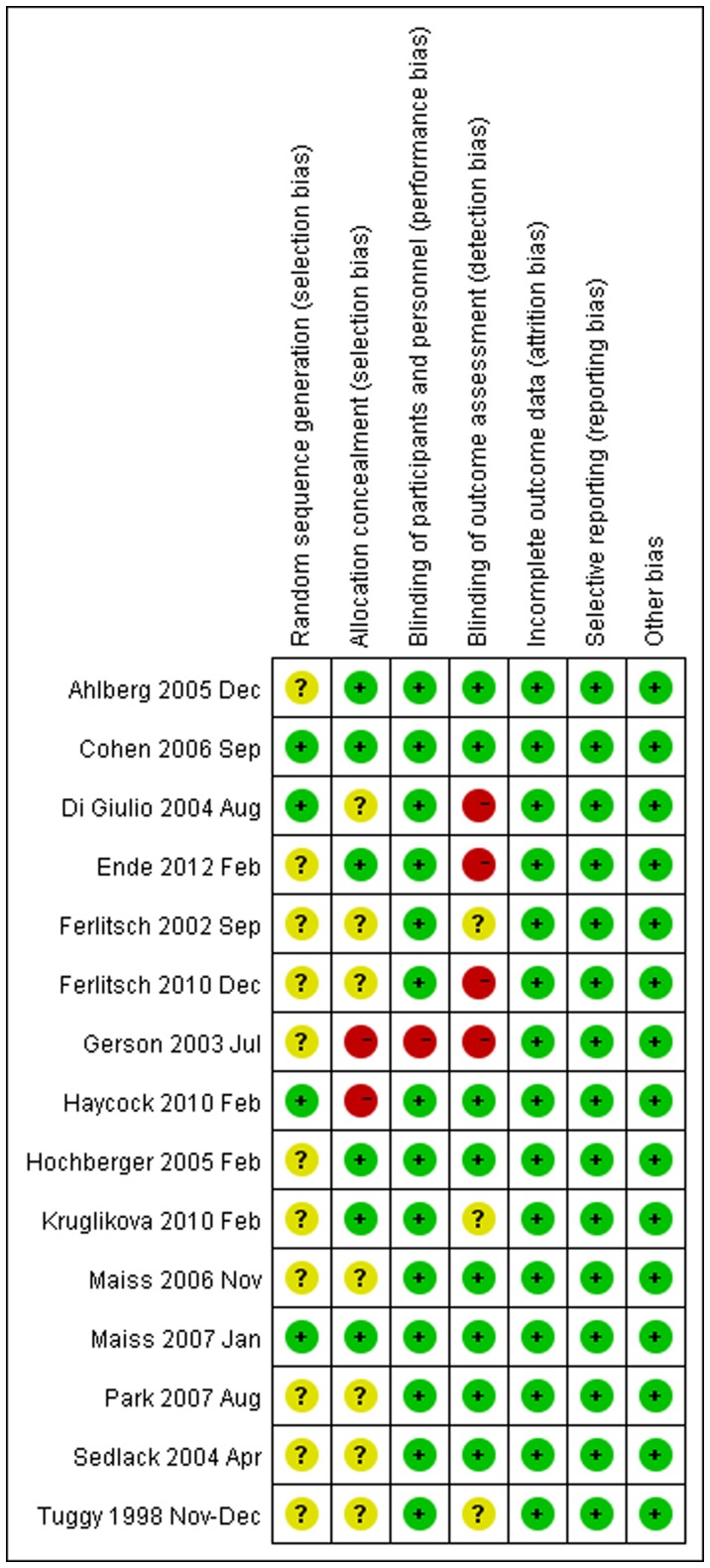
Risk of bias summary. Yellow circles, green circles, and red circles indicate “unclear risk of bias,” “low risk of bias,” and “high risk of bias,” respectively.

The risk of bias assessment is summarized in Figure 2 and Figure 3. Four studies were rated as high risk of bias due to blinding (Di Giulio 2004, Ende 2012, Ferlitsch 2010, Gerson 2003). Two studies were rated as high risk of bias for allocation sequence concealment (Gerson 2003, Haycock 2010).

### Effects of Interventions

#### Gastroscopy

**Figure 4:**
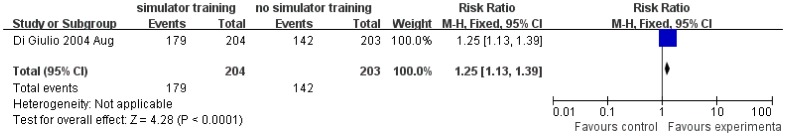
Forest plot of comparison: procedure completed independently for gastroscopy.

For gastroscopy training, virtual endoscopy simulator training was superior to control group for novices in independent procedure completion. Procedure completed independently was reported in 87.7% of participants in the simulator training group compared to 70.0% of participants in the no simulator training group (1 study, 22 participants, RR 1.25, 95% CI 1.13–1.39, *P*<0.0001; see Figure 4). A GRADE analysis (Table 1) indicated that the overall quality of the evidence for the primary outcome for procedure independent completion was low due to imprecision (i.e. only 1 study) and high risk of bias (i.e. instructors were not blinded as to whether trainees had or had not used the simulator.).

**Table 1:** GRADE analysis for main comparison for gastroscopy.

Simulator training versus bedside training for independent procedure completion for gastroscopy in novices					
**Bibliography:** Qiao W, Lv R. The effect of virtual endoscopy simulator training compared with conventional training for novices.					
**Outcomes**	**No of Participants (studies)** Follow up	**Quality of the evidence** (GRADE)	**Relative effect (95% CI)**	**Anticipated absolute effects**	
				
				**Risk with Bedside training**	**Risk difference with Simulator training** (95% CI)
**independent procedure completion for gastroscopy**	407 (1 study)	⊕⊕⊝⊝ **LOW** 1 ^,^ 2 due to risk of bias, imprecision	**RR 1.25** (1.13 to 1.39)	**Study population**	
				**700 per 1000**	**175 more per 1000** (from 91 more to 273 more)
				**Moderate**	
				**700 per 1000**	**175 more per 1000** (from 91 more to 273 more)

*The basis for the **assumed risk** (e.g. the median control group risk across studies) is provided in footnotes. The **corresponding risk** (and its 95% confidence interval) is based on the assumed risk in the comparison group and the **relative effect** of the intervention (and its 95% CI). **CI:** Confidence interval; **RR:** Risk ratio;

GRADE Working Group grades of evidence **High quality:** Further research is very unlikely to change our confidence in the estimate of effect. **Moderate quality:** Further research is likely to have an important impact on our confidence in the estimate of effect and may change the estimate. **Low quality:** Further research is very likely to have an important impact on our confidence in the estimate of effect and is likely to change the estimate. **Very low quality:** We are very uncertain about the estimate.

1 Instructors were not blinded as to whether trainees had or had not used the simulator.

2 Total number of events is less than 300.

**Figure 5:**
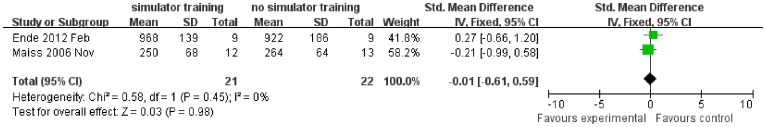
Forest plot of comparison: total procedure time (sec) for gastroscopy.

**Figure 6:**
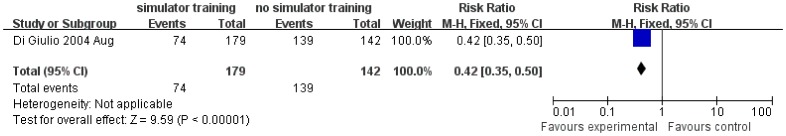
Forest plot of comparison: required assistance for gastroscopy.

There is no statistically significant difference in total procedure time for gastroscopy between the simulator group and no simulator group (2 studies, 43 participants, Std. Mean Difference 0.01, 95% CI −0.61–0.59, *P = *0.98). The *chi* ^2^ test showed no evidence of heterogeneity (*P* = 0.45) among the 2 trials comparing simulator training with conventional training. The *I* ^2^ statistic was 0% for this analysis, see Figure 5. The number of required assistance in simulator group is less than that in control group. Forty-one per cent of participants in the simulator group required assistance compared to 98% of participants in the no simulator group (1 study, 22 participants, RR 0.42, 95% CI 0.35–0.50, *P*<0.00001; see Figure 6).

#### Colonoscopy

**Figure 7:**
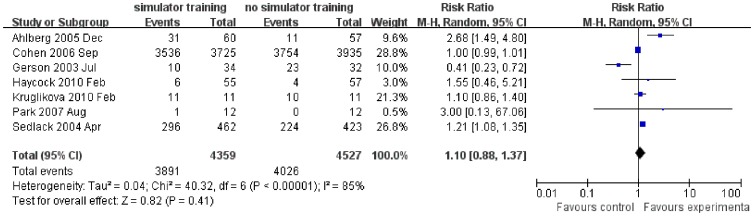
Forest plot of comparison: procedure completed independently for colonoscopy.

For colonoscopy training, there was no statistically significant difference in independent procedure completion between the simulator group and no simulator group. Procedure completed independently was reported in 89.3% of participants in the simulator training group compared to 88.9% of participants in the no simulator training group (7 study, 163 participants, RR 1.10, 95% CI 0.88–1.37, *P* = 0.41; see Figure 7). The *chi* ^2^ test showed great evidence of heterogeneity (*P*<0.00001) among the 7 trials comparing simulator training with conventional training. A randomized fixed-effect model was used for the *I* ^2^ statistic was 85% for this analysis. A GRADE analysis (Table 2) indicated that the overall quality of the evidence for supporting this outcome was low due to inconsistency (i.e. unexplained heterogeneity).

**Table 2:** GRADE analysis for main comparison for colonoscopy.

Simulator training versus bedside training for independent procedure completion for colonoscopy in novices					
**Bibliography:** Qiao W, Lv R. The effect of virtual endoscopy simulator training compared with conventional training for novices					
**Outcomes**	**No of Participants (studies)** Follow up	**Quality of the evidence** (GRADE)	**Relative effect (95% CI)**	**Anticipated absolute effects**	
				
				**Risk with Bedside training**	**Risk difference with Simulator training** (95% CI)
**independent procedure completion for colonoscopy**	8886 (7 studies)	⊕⊕⊝⊝ **LOW** 1 ^,^ 2 due to risk of bias, inconsistency	**RR 1.1** (0.88 to 1.37)	**Study population**	
				**889 per 1000**	**89 more per 1000** (from 107 fewer to 329 more)
				**Moderate**	
				**530 per 1000**	**53 more per 1000** (from 64 fewer to 196 more)

*The basis for the **assumed risk** (e.g. the median control group risk across studies) is provided in footnotes. The **corresponding risk** (and its 95% confidence interval) is based on the assumed risk in the comparison group and the **relative effect** of the intervention (and its 95% CI). **CI:** Confidence interval; **RR:** Risk ratio;

GRADE Working Group grades of evidence**High quality:** Further research is very unlikely to change our confidence in the estimate of effect. **Moderate quality:** Further research is likely to have an important impact on our confidence in the estimate of effect and may change the estimate. **Low quality:** Further research is very likely to have an important impact on our confidence in the estimate of effect and is likely to change the estimate. **Very low quality:** We are very uncertain about the estimate.

1 Neither the investigators nor the participating residents were blinded to the group assignment in Gerson 2003

2 Unexplained heterogeneity.

**Figure 8:**
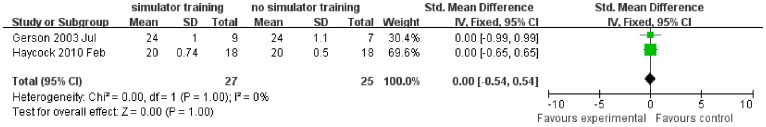
Forest plot of comparison: total procedure time (min) for colonoscopy.

**Figure 9:**
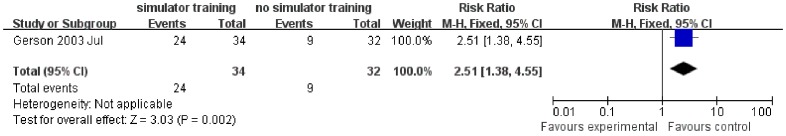
Forest plot of comparison: required assistance for colonoscopy.

There was no statistically significant difference in total procedure time for colonoscopy between the simulator group and no simulator group (1 study, 16 participants, Std. Mean Difference 0.00, 95% CI −0.99–0.99, *P* = 1.00; see Figure 8). The number of required assistance in simulator group is more than that in control group. Seventy per cent of participants in the simulator group required assistance compared to 28% of participants in the no simulator group (1 study, 16 participants, RR 2.51, 95% CI 1.38–4.55, *P* = 0.002; see Figure 9). Comparison of time to hepatic flexure, time to splenic flexure, time to cecum and visualized mucosa (%) for colonoscopy was tried, however, no enough extractable data could be found.

## Discussion

The results of this systematic review suggest that virtual endoscopy simulator training is effective for novices in gastroscopy, but not in colonoscopy. Virtual endoscopy simulator training is superior to control for procedure completed independently and required assistance in gastroscopy training. More research is needed to confirm the efficacy of simulator-based training method for endoscopy learners. Accordingly, virtual simulator training method could be considered as an alternative to conventional teaching for novices.

There is no statistically significant difference in independent procedure completion and total procedure time in colonoscopy training. The number of required assistance in simulator group is more than that in control group, which could be explained by the fact that the control group where not real novices. A 41 participants involved study training showed significant improvements from pretraining to posttraining in cecum intubation time (229±97 vs. 150±57 s; p<0.001), total time (454±147 vs. 320±115 s; p<0.001), and screening efficiency (85% ±12% vs. 91% ±5%; p<0.002) [28], but there is no comparison to any other training method. Consequently, additional trials are still needed to evaluate comparison of time to hepatic flexure, time to splenic flexure, time to cecum and visualized mucosa (%).

Patient discomfort and performance assessment by the experts are two important aspects for evaluation of simulator training. Increased patient comfort was found from simulation training, demonstrating that computer-based endoscopy simulator training has a direct benefit to the patient [29]. The Global Assessment of Gastrointestinal Endoscopic Skills (GAGES) Upper Endoscopy (GAGES-UE) and Colonoscopy (GAGES-C) developed by expert endoscopists, are easy to administer and adhere to and meet high standards of reliability and validity, which may contribute to the definition of technical proficiency in endoscopy [30]. However, it is difficult to compare simulator training with conventional training on patient discomfort and performance assessment for different points-scoring system used in different studies. A popular standardized points-scoring system is needed to establish. Further research comparing computer-based simulator training with traditional teaching is needed.

Simulator training method could not only used for basic manual skills, but also for therapeutic endoscopic skills. A single-blind, randomized, controlled trial compared the effect of knowledge-based teaching and simulator-based skills training in 4 therapeutic endoscopic procedure: control of nonvariceal upper GI bleeding, polypectomy, stricture dilation, and percutaneous endoscopic gastrostomy tube insertion. Simulator training group significantly improved performance of polypectomy, control of upper GI bleeding, and esophageal dilation [31]. Twenty-eight fellows in New York and 36 in France were trained for manual skills, injection, coagulation, hemoclip application and variceal ligation with the compact EASIE simulator. Successful hemostasis was significantly improved in performance of participants [32]. In a prospective multicenter randomized controlled trial during early training, a significantly higher proportion of the biliary cannulations performed by trainees with endoscopic retrograde cholangiopancreatography (ERCP) mechanical simulator practice were successful and with faster cannulation time compared with those performed by trainees without such practice [33]. Further studies comparing computer-based simulator training with traditional teaching for therapeutic endoscopic skills are needed.

Based on this systematic review, simulator training method has been shown to be effective for the training of beginners. To date the knowledge on the learning curves for residents, the endoscopy training curriculum, and the effects of tutor feedback on simulator training is very limited. Studies demonstrated that psychomotor training had a significant effect on the learning curves of a simulated colonoscopy [34 35]. Residents and nurses showed similar learning curve patterns. There were not significant differences between the groups in terms of volume of insufflated air, percentage of time without discomfort, and percentage of mucosa seen [36]. In 2001 a new training concept called “GATE- gastroenterological education- training endoscopy” was established, which provides a combination of background theory, video demonstrations, and simulator training. The integrated GATE training improved both theoretical knowledge and manual skill of physicians [37]. It seems that “no feedback, no learning”. A study demonstrated that in the absence of feedback, it is not possible to improve performance on the HT Immersion Medical Colonoscopy Simulator [38]. Above all, future studies are needed to assess different teaching methods for virtual simulator training.

Although, rigorous inclusion criteria has been made to reduce the heterogeneity. Several limitations of this systematic review should be noted. First, the presence of heterogeneity between studies is a concern. The included studies varied with respect to training schedule, training time, training procedure, type of simulator and teaching technique, which bring sources of heterogeneity [39]. Secondly, the systematic review relied on publications, not on individual patient data. At present, access to individual patient data is still very difficult, and a consensus should be reached that such data should be made available to address subsequent research questions. Thirdly, for colonoscopy training, novices in two studies (Ahlberg 2005, Sedlack 2004) had a formal gastroscopy training, which made it easier to learn colonoscopy. Additionally, the main source of bias comes from blinding of outcome assessment and allocation sequence concealment. We did not detect publication bias between studies, for there were too few studies included in each comparison to produce a meaningful analysis. These limitations all affect results.

## Conclusions

In summary, the limited data available suggest that simulator training might be effective for gastroscopy, but so far no data is available to support this for colonoscopy. Overall, the study population or trial size evaluated in studies of simulator training for endoscopy teaching is small. Well-designed randomized studies are needed to establish the optimal training curriculum for simulator method. Further studies comparing computer-based simulator training with traditional teaching for therapeutic endoscopic skills are needed. The efficacy of combination of simulator training and bedside teaching could also be evaluated in future studies.

## Supporting Information

Table S1 **Characteristics of the included studies.**
(XLSX)

Checklist S1 **The PRISMA checklist.**
(DOC)
